# Executive function and attention-deficit/hyperactivity disorder in Ugandan children with perinatal HIV exposure

**DOI:** 10.1017/gmh.2015.2

**Published:** 2015-05-08

**Authors:** M. D. Burkey, S. M. Murray, P. Bangirana, I. Familiar, R. O. Opoka, N. Nakasujja, M. Boivin, J.K. Bass

**Affiliations:** 1 Division of Child and Adolescent Psychiatry, Johns Hopkins School of Medicine, Baltimore, Maryland, USA; 2 Department of Mental Health, Johns Hopkins Bloomberg School of Public Health, Baltimore, Maryland, USA; 3 Department of Psychiatry, Makerere University College of Health Sciences, Kampala, Uganda; 4 Department of Psychiatry, Michigan State University, East Lansing, Michigan, USA; 5 Department of Pediatrics, Mulago Hospital/Makerere University College of Health Sciences, Kampala, Uganda; 6 Department of Neurology and Ophthalmology, Michigan State University, East Lansing, Michigan, USA

**Keywords:** Attention Deficit Disorder with Hyperactivity (MeSH), Executive Function (MeSH), Uganda (MeSH), perinatal HIV exposure

## Abstract

**Background.:**

Attention-deficit/hyperactivity disorder (ADHD) is among the most commonly diagnosed mental disorders in childhood and is associated with substantial deficits in executive functioning and lost academic and occupational attainment. This study evaluates symptoms of ADHD and their association with neurocognitive deficits in a cohort of rural Ugandan children who were born to HIV-infected mothers.

**Methods.:**

We assessed ADHD symptoms and executive function (including memory and attention) in a non-clinical sample of children born to HIV-infected mothers in rural eastern Uganda. Analyses included assessments of the psychometric properties, factor structure, and convergent and discriminant validity of the ADHD measure (ADHD-Rating Scale-IV); and executive function deficits in children meeting symptom criteria for ADHD.

**Results.:**

232 children [54% female; mean age 7.8 years (s.d. 2.0)] were assessed for ADHD and executive function deficits. The ADHD measure showed good internal consistency (α = 0.85.) Confirmatory factor analysis showed an acceptable fit for the diagnostic and statistical manual of mental disorders (DSM-5) two-factor model. Subjects meeting DSM-5 symptom criteria for ADHD had worse parent-rated executive function on six out of seven subscales.

**Conclusions.:**

Our results demonstrate structural validity of the ADHD measure with this population, strong associations between ADHD symptom severity and poorer executive function, and higher levels of executive function problems in perinatally HIV-exposed Ugandan children with ADHD. These findings suggest that ADHD may be an important neurocognitive disorder associated with executive function problems among children in sub-Saharan African settings where perinatal HIV exposure is common.

## Background

Over 60% of children in sub-Saharan Africa (SSA) are estimated to be at high risk for failing to reach their potential in cognitive development, with subsequent effects on educational attainment and poverty (Grantham-McGregor *et al*. 2007). The current study evaluates the importance of attention deficit/hyperactivity disorder (ADHD) – a common neurodevelopmental disorder (American Psychiatric Association, 2013) – among children living in SSA by assessing its association with executive function problems, an important and highly related domain of cognition (Diamond, 2013). A second goal of this paper is to address the lack of valid measurement tools for assessing child mental health problems in low-income country settings (Collins *et al*. 2011).

ADHD is a syndrome of hyperactivity, impulsivity, and impaired attention (American Psychiatric Association, 2013). Increasingly, ADHD is also recognized as a clinical problem related to underlying deficits in executive function (Willcutt *et al*. 2005; Mahone & Hoffman, 2007). Executive function is a developmental construct consisting of a combination of domains required for execution of purposeful actions; executive function has been defined as including attention control, cognitive flexibility, goal setting, and information processing (Anderson, 2002; Diamond, 2013). In their 2004 study, Biederman *et al*. identified higher rates of executive functioning problems in children with ADHD compared with non-ADHD controls (Biederman *et al*. 2004). Executive function deficits may be more prevalent among children with ADHD compared with other related child mental disorders. For example, compared with children with other externalizing disorders (e.g. oppositional defiant disorder and conduct disorder) but without ADHD, Clark *et al*. found that children with ADHD had increased impairment related to executive function deficits (Clark *et al*. 2000).

Through its association with impaired executive functioning, ADHD affects the basic cognitive skills necessary for academic success and social functioning. Longitudinal studies show ADHD to be a robust predictor of poor academic achievement, grade retention, low occupational attainment, and psychiatric and conduct problems into adulthood, including substance abuse and incarceration (Wilens *et al*. 1997; Leibson *et al*. 2001; Biederman *et al*. 2004; Biederman & Faraone, 2006). Given these significant long-term health consequences, ADHD's association with increased medical costs, a high estimated global prevalence, and the existence of efficacious and cost-effective treatments, the World Health Organization (WHO) has identified ADHD as one of two worldwide ‘priority (mental health) conditions’ of early childhood (World Health Organization, 2003).

SSA is an important setting in which to study ADHD and neurocognitive development. In SSA, high rates of HIV infection and cerebral malaria combined with the effects of severe poverty, including malnutrition and stunting, to substantially increase the risk of neurocognitive developmental delays among children (Grantham-McGregor *et al*. 2007; Beddington *et al*. 2008; Zeegers *et al*. 2010; Walker *et al*. 2011). High rates of poor cognitive and educational performance associated with these risk factors are proposed to be important factors in subsequent lost occupational attainment, high fertility, and intergenerational perpetuation of poverty (Grantham-McGregor *et al*. 2007). Executive function in particular appears susceptible to the kinds of environmental risks commonly found in SSA. A study in Mozambique showed that, among child development domains, language and executive function were most susceptible to the effects of extreme poverty (Fernald *et al*. 2011). Prior studies in Uganda have revealed specific deficits in the executive function domains of working memory and attention among children with cerebral malaria (Boivin *et al*. 2007).

SSA is also an important place to study child neurocognitive disorders due to the high prevalence of HIV and poverty (Grantham-McGregor *et al*. 2007). Theoretical and empirically observed risks to neurocognitive development are related to direct toxic effects of HIV (in infected children) and antiretroviral medications (Blanche *et al*. 1999) on the developing brain, as well as the multiple social–environmental risks that commonly accompany maternal HIV infection (e.g. poverty, orphanhood, stressful life events) (Mellins & Malee, 2013; Llorente *et al*. 2014). Children with HIV infection and perinatal HIV exposure but no infection have been shown to exhibit high rates of emotional and behavioral problems, including hyperactivity, impulsivity, and inattention (Mellins *et al*. 2003; Nozyce *et al*. 2006; Mellins & Malee, 2013). The largest study of ADHD in HIV-infected children in SSA showed a very high prevalence (>24%) of all subtypes of ADHD, with duration of antiretroviral therapy a risk factor for greater severity of ADHD symptoms (Zeegers *et al*. 2010). One of the few studies evaluating ADHD in children with perinatal HIV exposure found high rates of ADHD in children (drawn from medical clinics in New York City) with (21.8%) and without (11.6%) HIV infection (Mellins *et al*. 2012).

While there are few studies of executive function in children with perinatal HIV exposure, a moderately sized observational study in Thailand and Cambodia demonstrated intellectual and memory deficits in HIV-exposed but uninfected children compared with unexposed children (Kerr *et al*. 2014). Another study found greater executive function deficits in HIV-infected children compared with uninfected children; the differences were explained by moderating treatment and psychosocial variables (Llorente *et al*. 2014). Taken together, children with perinatal HIV exposure represent a high-risk group for neurodevelopmental delays, especially when living in situations characterized by poverty, HIV-related stigma, and limited educational opportunities.

Despite WHO's recognition of the importance of ADHD globally and the presence of prevalent overlapping risk factors for neurocognitive delay in SSA, ADHD is often considered a disorder limited to high-income countries. A large, multi-national systematic review estimated the world-wide prevalence of ADHD in community settings at 5.3% (Polanczyk *et al*. 2007). Although the review included 102 studies, only four were conducted in Africa (Polanczyk *et al*. 2007). In a 2012 systematic review of ADHD studies in African settings, Bakare (2012) identified only nine qualifying epidemiologic studies. In Bakare's survey, community- and school-based samples demonstrated ADHD prevalence between 1.5% and 8.7%, whereas clinical populations of children with histories of HIV, tuberculosis, meningitis, and intellectual disability found rates ranging from 45% to 100% (Bakare, 2012). In Uganda, the site of the current study, we are only aware of one study related to ADHD has previously been published. In this paper, Idro *et al*. (2010) described transient ADHD-like symptoms following cerebral malaria infection. Despite epidemiologic evidence suggesting similar rates of ADHD across geographic settings, questions remain about the transferability of the ADHD construct to non-American or non-Western settings (Faraone *et al*. 2003).

To address questions about the relevance of ADHD to children in African settings this study evaluated ADHD symptom patterns and executive function problems associated with ADHD symptoms in a group of school-age children living in rural Uganda. The sample for this study was anticipated to be at high risk for neurocognitive impairment (including executive function problems and ADHD) due to previously reported risk factors of being born to HIV-infected mothers and living in a region with high rates of poverty where malaria is endemic. While we anticipated high rates of ADHD and executive function problems in this sample, an accurate estimate of prevalence of these problems was beyond the scope of the current study. Instead, the primary aim of this study was to address the broader question of whether ADHD symptoms were associated with important and expected impairments in cognitive function (i.e. executive function problems) among Ugandan children with *in utero* HIV exposure. The secondary aim was to assess the construct validity and psychometric properties of a common ADHD measurement in our sample. The secondary aim addresses the programmatic concern of a paucity of validated instruments for child mental health in SSA and the conceptual question of to what extent a previously validated (in other settings) ADHD instrument measures a similar and coherent construct (Thompson & Daniel, 1996) in a distinct socio-cultural context.

While behavioral rating scales are commonly used in the assessment of ADHD, there is little information available about the cross-cultural validity of such tools in SSA settings. To assess ADHD symptoms, we selected the ADHD-Rating Scale IV (DuPaul *et al*. 1998), a DSM-based parent-report scale that has shown good cross-cultural reliability and construct validity, albeit with little data from SSA. We hypothesized that ADHD symptom scores would be internally consistent and demonstrate a two-factor structure, similar to Western reference populations.

Based on previous studies of ADHD and executive function (Mahone *et al*. 2002), we hypothesized that ADHD symptom scores would be strongly associated with parent-reported executive function problems (similar construct, similar method), but would associate less strongly with performance-based assessments of inattention and impulsivity (similar construct, different method), and would not be associated with performance-based measures of general cognitive function (different construct, different method).

## Methods

We collected parent-report and performance-based measures of ADHD symptoms and neurocognitive functioning in a sample of children in rural Uganda. This study was approved by the Michigan State University Biomedical IRB and Makerere University Research and Ethics Committee (Uganda). Research permission was issued by the Ugandan National Council for Science and Technology.

### Study setting and subjects

This study was conducted in a community (i.e. non-clinical) setting in rural eastern Uganda. Uganda is a low-income country in East Africa that is home to 37.6 million people (The World Bank, 2014). Uganda has a limited mental health workforce with only 0.09 psychiatrists per 100 000 population, the vast majority of whom practice in urban areas (World Health Organization, 2011). In the study region, malaria is endemic, HIV is common, and rates of malnutrition are high.

Subjects for this study were recruited from an ongoing trial that includes caregivers and children ages 2–5 years old for a study of a caregiver training intervention on child neurocognitive development. Participants lived within a 20 km radius of Tororo town and were referred by the Infectious Disease Research Collaboration at Tororo District Hospital where the mothers had previously been evaluated and/or treated for HIV. As part of a supplemental study, up to four other children in each study home, ages 0–12, were recruited to be regularly assessed on similar measures to the parent study to identify potential dispersion of intervention effects to other children in the home. The subjects in the current study came from the supplemental study and include children between 3 and 12 years. All of the study children were born to HIV-infected mothers. The children's HIV status was not directly assessed as part of this study; however, the use of prophylaxis for opportunistic infections was available as a proxy and was 95% predictive of HIV infection status in a related study in the same community. Written informed consent was obtained from the child's primary caregiver and assent was obtained from children aged 7 and older.

Interviews were conducted by Ugandan researchers in the participants’ homes and/or the study office. All interviewers had completed at least bachelor's level academic training. Interviewers received two 1-week trainings by the study authors on caregiver assessments (focused on interviewing and scoring; trainer: JB) and child neurodevelopmental performance tests (focused on implementation and scoring; trainers: PB and MB) before data collection started.

Exclusion criteria for caregivers from the parent study included current suicidality or psychosis. Exclusion criteria for study children included: history of serious birth complications, severe malnutrition, bacterial meningitis, encephalitis, cerebral malaria, or other known brain injury or disorder requiring hospitalization.

### Measurements

Baseline data were collected on demographics, clinical (health) characteristics, family characteristics, the Home Observation for Measurement of the Environment (HOME) (Caldwell & Bradley, 1984), ADHD symptoms using the ADHD-RS-IV (DuPaul *et al*. 1998), executive function as assessed using the Behavior Rating Inventory of Executive Function (BRIEF) (Gioia *et al*. 2000), attention as assessed by continuous performance tasks using the Test of Variables of Attention (TOVA) (Greenberg & Waldmant, 1993), and general cognitive ability as assessed by the Kaufman Assessment Battery for Children—2nd edition (KABC-II) (Kaufman, 2004). All study assessments were translated into the local study languages: Japadhola, Ateso, and Luganda. Standardized translation procedures included: translation by a bi-lingual translator, piloting, and revision with a focus group representative of the study population (repeated as necessary until comprehension was achieved), back-translation to English and checking by two study investigators. The authors of the BRIEF also reviewed and approved all back-translations for the BRIEF. Modifications to the instruments mostly involved changes to idiomatic phrases, explanation of unfamiliar concepts, and replacement of terms for unfamiliar objects. (e.g., Japadhola does not have direct translations for ‘daily routine’ or ‘desk’ – benches are used in classrooms.)

The primary caregivers (91% mothers) were the informants for the ADHD-RS-IV and BRIEF and the children completed the KABC-II and TOVA. Parent report measures (ADHD-RS-IV and BRIEF) were collected by reading the questionnaire items to the caregivers, word by word. In case of lack of understanding, the interviewer was instructed to repeat the item to the caregiver as many times as necessary. In very few cases, caregivers did not understand in spite of repeated attempts and clarification was used to put the item in context. The ADHD-RS-IV and BRIEF (preschool or standard versions) were administered for all children, whereas the TOVA and KABC-II were administered only to children aged >5. All measurements were made prior to initiation of the caregiver training intervention.

### ADHD-Rating Scale IV

ADHD symptoms were assessed using parent report on the ADHD-RS-IV, an 18-item scale that assesses the presence and severity of DSM-IV symptoms of ADHD, including a nine-item subscale for inattention and a nine-item subscale for hyperactivity/impulsivity (DuPaul *et al*. 1998). Scores on the ADHD-RS-IV range from 0 to 36 with higher scores corresponding to a larger number or greater intensity of symptoms. The ADHD-RS-IV has good test-retest reliability, established validity in cross-cultural settings, and good discriminative validity (e.g. compared with measures of disruptive behavior problems) (Zhang *et al*. 2005; Döpfner *et al*. 2006). The ADHD-RS-IV has also demonstrated good reliability and concurrent validity with the Conners Teacher Rating Scale in pre-school age children (McGoey *et al*. 2007).

### Behavior rating inventory of executive function

To assess executive functioning, we employed the BRIEF school-age version (ages 5–12) (Gioia *et al*. 2000) and BRIEF-Preschool version (BRIEF-P) (ages 3–5) (Gioia *et al*. 2003). The BRIEF is an 86-item parent-report rating scale used to assess five theoretically and statistically derived domains of executive functioning. Children with ADHD have been found to score significantly higher on all five primary indices of the BRIEF-P (Mahone *et al*. 2002; Mahone & Hoffman, 2007). On the BRIEF, indices of greatest interest for concurrent validity [based on Mahone *et al*. (2002) evaluation of the BRIEF in ADHD and non-ADHD children] included: inhibit scale, emotion control scale, and working memory scale. In order to combine pre-school and school-age versions of the BRIEF, we utilized *t*-scores to assess relative deviation from age-normed values. The back-translation of the final version of the BRIEF was approved by one of the test authors (Peter Isquith).

### Test of Variables of Attention

TOVA (Greenberg & Waldmant, 1993) is a computerized go/no-go test that utilizes visual and auditory stimuli to measure errors of commission and omission and response time. These metrics are combined into a composite ‘TOVA ADHD Score’. On the TOVA, children with ADHD have been found to have slow reaction times with high variability (Harris *et al*. 1995). Other evaluations using the TOVA have found good sensitivity, but low specificity for correctly identifying patients with ADHD (Lovejoy *et al*. 1999). The TOVA has previously been used in SSA to assess attention among children with a history of cerebral malaria (Boivin, 2002) and in Uganda among HIV-infected children (Boivin *et al*. 2010) and healthy control children (Ruel *et al*. 2012). Ruel *et al*. (2012) demonstrated worse reaction times and visual ADHD scores on the TOVA among children with HIV compared with HIV-uninfected children.

### KABC-II

KABC-II is a paper-and-pencil assessment tool for general cognitive ability in children ages 3–18 (Kaufman, 2004). The KABC-II's subtests minimize verbal instructions and responses in an effort reduce cultural biases in the assessment of cognitive ability. The Mental Processing Index on the KABC-II is a composite measure of general cognitive ability (with higher numbers indicating greater ability) and the KABC-II's Number Recall test assesses short-term memory (a component of executive function).

### Statistical methods

Statistical analyses were undertaken to assess the internal consistency and factor structure of the ADHD-RS-IV, convergent and discriminant validity, prevalence of and risk factors for ADHD, and executive function deficits by ADHD status in the study population. Internal consistency was assessed using Cronbach's alpha. For factor analysis, we first used principal components analysis to extract initial factors and identify the number of factors to retain. Outcomes of exploratory factor analysis depend heavily on the number of factors retained and there are no established criteria to determine this procedure, so a number of decision rules were applied examining several solutions before coming to a final conclusion. The decision rules we used included: assessing the eigenvalues (selecting factors with a value above 1); graphically representing the eigenvalues to visually analyze the relative importance of the factors, where a sharp drop in the plot signals that subsequent factors are ignorable (scree plot); and using parallel analysis to adjust for sample bias in the observed eigenvalues (Dinno, 2009). Exploratory and confirmatory factor analyses (using the DSM-5 two-factor solution of inattention and hyperactivity/impulsivity) were then performed and compared with previous findings from international samples.

The primary assessment of convergent validity was association between ADHD-RS-IV total symptom score and the BRIEF ‘inhibit’ scale [based on theoretical formulations and empirical data supporting inhibition of inappropriate thoughts or behaviors as a primary deficit in ADHD (Mahone *et al*. 2002)]. Secondary measures of convergent validity were associations between ADHD-RS-IV total score and other measures of executive function, including: (1) other BRIEF global indices of executive function; (2) TOVA ADHD Score, TOVA total percent commission errors, TOVA total omission errors, and TOVA response time variability; and (3) KABC-II Number Recall. The primary measure of discriminant validity was association between ADHD-RS-IV total symptom score and KABC-II Mental Processing Index.

To assess the relationship between ADHD and executive function, we used mixed effects linear regression models with ADHD-RS-IV total score as the dependent variable and the BRIEF indices and scales and the TOVA scores as independent variables. We also used mixed effects linear regression models to assess for differences in cognitive function by ADHD symptom level, using the KABC-II Mental Processing Index as the independent variable. We assessed whether children meeting ADHD criteria had greater deficit-level executive dysfunction (*t*-score ≥65) using χ^2^ analyses. As multiple children from the same caregiver were eligible for the study, we assessed the degree of clustering effects by family (e.g. due to heritability and use of the same rater on parent-report forms) by examining intraclass correlation coefficients. Since intraclass correlation within families was found to be high, we included shared primary caregivers as a random effect.

Mixed effects logistic regression was used to assess risk factors for binary outcomes. ADHD prevalence was estimated using standard DSM-5 symptom criteria (American Psychiatric Association, 2013) based on ADHD-RS-IV scoring conventions (DuPaul *et al*. 1998). Acceptable level of statistical significance was pre-specified as *p* = 0.05 (two-sided). Data analysis was performed using Stata 12.0 (Stata Corporation, 1985–2013).

## Results

### Subjects

Two hundred and thirty two children were assessed; the mean age of the study sample was 7.8 (s.d. 2.0) years and 54% of the participants were female. Table 1 presents the demographic and clinical characteristics of the study sample and summarizes their scores on the ADHD-RS-IV and ADHD by sub-type. The mean score on the ADHD-RS-IV was 12.7 (s.d. 8.6). Using DSM-5 symptom criteria cutoffs, the proportion of children in our sample meeting criteria for ADHD (including all subtypes) was 5.6% (*n* = 13). The proportion of children with each ADHD subtype was: predominantly inattentive, 2.6%; predominantly hyperactive/impulsive, 0.9%; and combined (i.e. meeting criteria for *both* inattentive and hyperactive/impulsive subtypes), 2.2%. Of note, these categorical estimates of ADHD should be interpreted with caution since clinical cutoffs for the ADHD-RS-IV have not been established in Uganda.

**Table 1.** Summary of baseline characteristics and ADHD measures (n = 232)

Variable	*n* (%) or Mean (s.d.)
Female sex	125 (53.9%)
Age	
3–5	16 (6.9%)
6–9	143 (61.6%)
10–12	73 (31.5%)
Primary caregiver	
Mother	210 (90.5%)
Grandmother	14 (6.0%)
Aunt	3 (1.3%)
Step-mother	4 (1.7%)
Sibling	1 (0.4%)
Born >1 month pre-term	40 (17.2%)
History of malaria	227 (97.8%)
HIV (proxy variables)	
On HAART	10 (4.3%)
On opportunistic infection prophylaxis	19 (8.2%)
Body mass index	14.1 (1.5)
Mean ADHD-RS-IV scores	
Total score	12.3 (8.6)
Inattention score	6.23 (4.8)
Hyperactivity/impulsivity score	6.07 (4.6)
ADHD prevalence	
ALL subtypes	13 (5.6%)
Combined subtype	5 (2.2%)
Inattentive only	6 (2.6%)
Hyperactive/impulsive only	2 (0.9%)

### Psychometric properties and factor analysis of ADHD-RS-IV

**Fig. 1.**
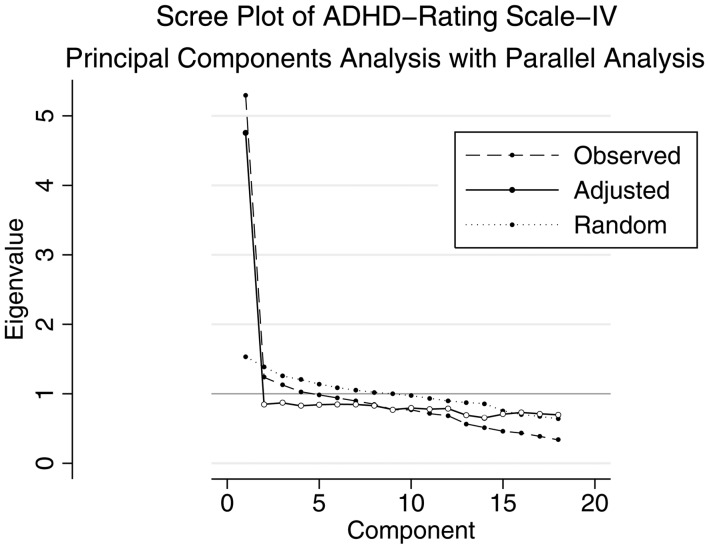
Scree plot of eigenvalues after principal components analysis for the ADHD-RS-IV. This figure illustrates the unadjusted and adjusted eigenvalues (*y*-axis) of each principal component (*x*-axis) for the ADHD-RS-IV. The dotted line (‘Random’) indicates the expected eigenvalues derived from a random dataset in parallel analysis.

Cronbach's alpha for the ADHD-RS-IV was 0.85 for the full scale, 0.75 for the inattention subscale, and 0.73 for the hyperactivity/impulsivity subscale. [DeVellis (2011) suggests that scores above 0.70 are ‘respectable’ and above 0.80 are ‘very good’.] There was no significant improvement to Cronbach's alpha when any individual items were dropped. Principal components analysis identified four factors with eigenvalues >1 (Factor 1: 5.29, 2: 1.24, 3: 1.13, 4: 1.03, 5: 0.98), while visual inspection of the scree plot (Fig. 1) showed a sharp shoulder after the first principal component, suggesting a one-factor solution. Using a parallel analysis (Dinno, 2009) we similarly found that a one-factor solution provided the best fit (Fig. 1). Since using eigenvalues alone has been shown to over-extract factors (Costello & Osborne, 2005) and parallel analysis has been shown to be among the most accurate methods for factor retention decisions (Dinno, 2009), we continued with a one-factor solution. The single factor explained 84% of the covariance. Items that loaded most strongly on the factor included ‘forgetful’, ‘interrupts’, and ‘on the go’ and the least strongly included ‘blurts out answers’, ’ (has) difficulty sustaining attention to tasks’, and ‘difficulty playing quietly’.

In confirmatory factor analysis, the standard two-factor solution (inattention and hyperactivity/impulsivity) showed acceptable fit indices [RMSEA = 0.059 (acceptable fit); CFI = 0.882 (poor fit); SRMR = 0.052 (acceptable fit)], suggesting plausibility of a two-factor model of ADHD. Measures of comparative fit suggested that the two-factor model was superior to the one-factor model (Akaike Information Criterion: 9567 *v.* 9571; Bayesian Information Criterion: 9746 *v.* 9750; CFI: 0.882 *v.* 0.877, respectively.)

### Associations between ADHD symptoms and executive function

Greater total ADHD symptom scores were strongly associated with higher levels of parent reported executive function problems on the BRIEF (*p* < 0.001 for all scales and indices, including Inhibit scale, Global Executive Composite, Working Memory scale, and Metacognitive Index) (Table 2). All associations remained significant at the *p* < 0.001 level after adjusting for child age and sex (which were both associated with executive function deficits). Higher total ADHD symptom scores were also associated with worse performance-based measures of executive function on the TOVA (*p* = 0.01–0.04), but not with the TOVA ADHD Index (*p* = 0.92) (Table 3). Total ADHD symptom scores were not significantly associated with KABC-II Number Recall (*p* = 0.14) (Table 3). Total ADHD symptom score was not significantly associated with general cognitive function as measured by the KABC-II MPI (*p* = 0.15)

**Table 2.** Mixed effects estimate^a^ of association between BRIEF^b^ and ADHD-RS-IV ratings (n = 231)

BRIEF scale	ADHD-RS-IV	ADHD-RS-IV	ADHD-RS-IV
	Total score β coefficient. (*p* value)	Hyperactive/Impulsive. score β coefficient. (*p* value)	Inattention score β coefficient. (*p* value)
Scales			
Working memory	0.44 (<0.001)	0.20 (<0.001)	0.24 (<0.001)
Inhibit	0.47 (<0.001)	0.22 (<0.001)	0.22 (<0.001)
Shift	0.34 (<0.001)	0.18 (<0.001)	0.18 (<0.001)
Emotion control	0.31 (<0.001)	0.16 (<0.001)	0.15 (<0.001)
Plan/organize	0.29 (<0.001)	0.13 (<0.001)	0.16 (<0.001)
Indices (emerging)			
Metacognitive index^c^	0.39 (<0.001)	0.19 (<0.001)	0.21 (<0.001)
General executive composite	0.48 (<0.001)	0.23 (<0.001)	0.24 (<0.001)

a Mixed effects models included clustering by primary caregiver

b Data from the BRIEF were combined as *t*-scores from the BRIEF-preschool version (ages 3–5) and standard (school-age) BRIEF (ages 5–12).

c Data are combined from the ‘Emerging Metacognitive Index’ on the BRIEF-Preschool version and the ‘Metacognitive Index’ on the school-age (standard) form of the BRIEF.

**Table 3.** Mixed effects estimate*^a^* of association between performance tests and ADHD-RS-IV scores (n = 212)*^b^*

Performance test	ADHD-RS-IV	ADHD-RS-IV	ADHD-RS-IV
	Total score β coefficient (*p* value)	Hyperactive/impulsive score β coefficient (*p* value)	Inattention score β coefficient (*p* value)
KABC-II			
KABC number recall	−0.36 (0.14)	−0.20 (0.15)	−0.10 (0.50)
KABC mental processing index	−0.09 (0.15)	−0.06 (0.10)	−0.03 (0.43)
TOVA			
TOVA ADHD score	0.02 (0.92)	0.02 (0.78)	−0.002 (0.98)
TOVA total commission errors	0.08 (0.04)	0.04 (0.06)	0.05 (0.03)
TOVA total omission errors	0.05 (0.02)	0.03 (<0.01)	0.01 (0.30)
TOVA total response time	0.01 (0.02)	0.003 (0.04)	0.003 (0.05)
TOVA response time variability	0.01 (0.01)	0.007 (0.02)	0.006 (0.05)

a Mixed effects models were adjusted for clustering by primary caregiver.

b KABC-II and TOVA evaluations were performed only on the subgroup of participants age 5 and over.

### ADHD risk factors

Multilevel mixed-effects logistic regression analysis did not reveal significant associations between ADHD and any of the individual or family-level risk factors assessed. Factors evaluated included: sex, age, HIV proxy variables (i.e. on HAART or on opportunistic infection prophylaxis), anthropometric measures (*z*-score for height, MUAC), having a non-(biological) parent caregiver, both parents unemployed, total HOME score, and harsh parenting (an index of five items from the HOME related to harsh/punitive parenting practices). There were no significant associations between either opportunistic infection prophylaxis and ADHD (*p* = 0.44) or HAART and ADHD (*p* = 0.68). Of note, we were unable to assess odds ratios for history of malaria or preterm birth, since all children meeting ADHD symptom criteria had been exposed to malaria and none of the ADHD cases were born preterm.

To further investigate ADHD risk factors in this novel study setting, we conducted two additional sensitivity analyses (all accounting for clustering by shared primary caregiver) using: (1) an alternate categorical definition with subjects scoring in the upper 10th percentile on the ADHD-RS-IV classified as ‘cases’ (i.e. total score > 22; *n* = 27), and (2) continuous ADHD symptom scores as the dependent regression variable. In the first analysis, there were no individual or family level risk factors. In the second analysis, age, MUAC, and height *z*-score were significantly associated with ADHD symptom scores, whereas sex and HIV proxy variables remained non-significant. In adjusted regression models (including age, MUAC, and height *z*-score), only age remained significantly associated with ADHD symptom scores.

### Executive function problems in children with ADHD

Children meeting symptom criteria for ADHD had significantly higher mean problem scores in all domains of executive functioning as measured by the BRIEF (see Table 4). In addition, they were more likely to score in the deficit range (*t*-score ≥65) on all indices and scales of the BRIEF (*p≤*0.001 for all comparisons) (results not shown). There were no significant differences in performance tests of executive function between children who met symptom criteria for ADHD and those who did not. These findings remained consistent in a sensitivity analysis defining children in the upper 10^th^ percentile of ADHD-RS-IV scores as ‘cases’.

**Table 4.** Comparison of characteristics and executive function measures between ADHD v. non-ADHD subjects

	Overall sample (*n* = 232)	ADHD (*n* = 13)	Non-ADHD (*n* = 219)	*β* coefficient. from logistic regression (*p* value)*
	Mean (s.d.)	Mean (s.d.)	Mean (s.d.)	
Age	7.8 (2.0)	7.8 (2.2)	7.8 (2.0)	−0.29 (0.37)
Sex (% females)	125 (53.9%)	9 (69.2%)	116 (53.0%)	0.69 (0.33)^d^
BRIEF scales/indices^a^				
Working memory	54.8 (10.6)	72.5 (12.1)	53.8 (9.5)	0.18 (<0.001)
Inhibit	51.0 (11.4)	69.9 (13.0)	49.8 (10.3)	0.16 (0.02)
Shift	54.1 (11.4)	68.2 (15.6)	53.3 (10.5)	0.10 (<0.001)
Emotion control	55.5 (12.9)	68.1 (15.0)	54.7 (12.4)	0.08 (<0.01)
Plan/organize	56.4 (12.2)	71.9 (12.0)	55.5 (11.6)	0.12 (0.001)
(Emerging) metacognition index	53.1 (12.1)	71.3 (13.0)	52.0 (11.2)	0.15 (0.11)
Global executive composite	53.4 (11.8)	73.0 (14.4)	52.2 (10.5)	0.16 (<0.001)
TOVA scales^b^				
TOVA ADHD score	−2.85 (3.6)	−0.93 (3.1)	−2.97 (3.58)	0.17 (0.04)
TOVA total commission errors (%)	13.3 (12.9)	13.4 (14.7)	13.3 (12.8)	0.008 (0.88)
TOVA total omission errors (%)	24.3 (24.3)	11.2 (17.1)	25.1 (24.4)	−0.04 (0.14)^d^
TOVA response time (ms)	699.0 (175.3)	682.6 (137.6)	700.0 (177.5)	−0.0006 (0.67)
TOVA response time variability	263.9 (99.4)	232.5 (27.1)	265.8 (7.0)	−0.004 (0.27)
KABC scores^b^			
Mental Processing index	32.9 (9.1)	32.8 (7.7)	32.9 (9.2)	0.01 (0.86)
Number recall	6.4 (2.1)	6.75 (1.5)	6.4 (2.2)	0.08 (0.40)^d^
Sequential processing scale^c^	73.0 (10.4)	74.8 (8.0)	72.9 (10.5)	0.02 (0.42)^d^
Simultaneous processing scale^c^	64.0 (9.4)	62.0 (7.3)	64.1 (9.5)	−0.03 (0.32)^d^
Planning/reasoning scale^c^	59.7 (7.1)	59.9 (6.4)	59.7 (7.1)	0.004 (0.93)
Learning ability scale^c^	68.0 (10.2)	67.7 (10.7)	68.0 (10.2)	0.04 (0.58)
Delayed recall scale^c^	69.4 (10.0)	71.2 (9.3)	69.3 (10.1)	0.02 (0.34)^d^
Non-verbal index^c^	58.4 (9.1)	57.1 (10.0)	58.5 (9.0)	0.003 (0.97)

a BRIEF scales and indices presented as t-scores.

b N for TOVA & KABC: ADHD (*n* = 12), non-ADHD (*n* = 200) as only children age 5 and over were assessed.

c Values indicated for these scales and indices are standard scores.

d Indicates standard regression models adjusted for clustering by primary caregiver; used when mixed-effects models failed to converge.

* *p* values for mixed effects logistic regression models included clustering by primary caregiver.

## Discussion

Our study demonstrated strong associations between ADHD symptom severity on the ADHD-RS-IV and greater executive function problems in Ugandan HIV-affected children. Children meeting symptom criteria for ADHD were significantly more likely to exhibit deficit-level impairments in all measured domains of executive function on parent-reported measures. Overall, the findings of this study are comparable with studies in the USA and elsewhere demonstrating significant executive dysfunction in children with ADHD (Mahone *et al*. 2002). Therefore, our findings provide preliminary support to arguments that ADHD is a neurodevelopmental disorder that is likely to be relevant to SSA settings given its association with expected impairment and the coherent factor structure of parent-reported ADHD symptoms.

To the best of our knowledge, this study is the most comprehensive evaluation of executive function in ADHD in SSA to-date, and is the first to evaluate this subject in children with perinatal HIV exposure. Given the prevalent and wide-ranging neurodevelopmental difficulties of children with HIV infection and perinatal exposure that commonly include ADHD-type symptoms (Mellins *et al*. 2003; Wolters *et al*. 2005), developing valid assessment tools, accurate epidemiologic assessments, and a better understanding of disorder burden are urgent priorities for global mental health (Collins *et al*. 2011).

The instrument used to assess ADHD in this study – the ADHD-RS-IV – demonstrated good construct validity and psychometric properties. There was good internal consistency and a factor structure comparable with international reference groups, DSM-5 subtypes (American Psychiatric Association, 2013) and previous multinational studies using the same instrument (Zhang *et al*. 2005). The ADHD-RS-IV also showed good convergent validity with standard neuropsychological measures of executive function, including visual performance tasks. Our findings of greater executive function problems on the BRIEF and slower and more variable responses on the TOVA in children with symptoms of ADHD are consistent with previous findings in clinical samples (Harris *et al*. 1995; Mahone *et al*. 2002). The finding of no association between ADHD symptom scores and measures of general cognitive ability on the KABC-II supports the discriminant validity of the ADHD-RS-IV.

While our study was not designed to provide population prevalence estimates, a similar proportion of subjects in our study met criteria for ADHD compared with the community prevalence rate estimated in a large international meta-analysis (Polanczyk *et al*. 2007). Our estimate is lower than those described in previous studies in Africa (including the ones included in the above-noted meta-analysis), which have reported widely varying prevalence estimates (Kashala *et al*. 2005; Chinawa *et al*. 2014) with a mean near 8% (Polanczyk *et al*. 2007). We are not aware of any previous evaluations of ADHD in perinatally HIV-exposed but uninfected children in SSA.

Our finding that the proportion of children in our study who met ADHD symptom criteria was similar to international prevalence rates does not appear to support the idea that HIV-exposed children in SSA represent a high-risk population for neurodevelopmental disorders. However, there are two important factors that limit our ability to accurately estimate ADHD prevalence in this study. First, our sample was not randomly selected and therefore prevalence estimates cannot be accurately estimated for the underlying population. Second, clinical cutoffs for the ADHD-RS-IV have not been established in this setting; therefore, categorical diagnoses are likely to be systematically over- or underestimated. In contrast, the poorer scores on the BRIEF and KABC relative to norms in the USA (see Table 4) suggest that executive function and general cognitive function were relative areas of weakness in our high-risk sample. However, the possibility of measurement differences on these instruments also emphasizes the need for local normative data in order to more definitively understand the burden of neurocogntive delays in this setting. Therefore, future studies could build on our work and establish the prevalence of ADHD and executive function problems and the role of HIV exposure in similar populations by: (1) establishing clinical cutoffs for the ADHD-RS-IV (which this study shows has good construct validity and psychometric properties); (2) incorporating population-based sampling (including a comparison group of children not exposed to HIV); and (3) establishing normative data for neuropsychological instruments.

In contrast to clinical samples that report two to three times as many boys than girls have ADHD (Willoughby, 2003), our study found a slightly higher proportion of girls than boys met criteria for ADHD, though this difference was not statistically significant. This finding could partly be accounted for the differences between referred (clinical) and non-referred (community) samples, where gender differences are occasionally found to be negligible (McGee & Feehan, 1991).

A limitation of this study was that our diagnosis of ADHD relied on parent-report instrument since a trained child psychiatrist was not available to conduct clinical interviews. However, the instrument used has previously demonstrated excellent psychometric and construct and criterion validity across settings, including in studies of executive function (Mahone & Hoffman, 2007) and in a multi-national study including patients from South Africa (Zhang *et al*. 2005). Furthermore, in this study population, the ADHD-RS-IV demonstrated very good performance on key measures of reliability and construct validity, including convergent and discriminant validity.

A second limitation was that the statistical power to assess differences between ADHD and non-ADHD patients was limited by the small number of subjects meeting criteria for ADHD. A related limitation was the limited variability in our malaria exposure variable and HIV infection proxy variables (i.e. >97% reported a history of malaria and fewer than 10% were on HAART or prophylaxis for opportunistic infections) that precluded an accurate calculation of malaria or HIV status as risk factors for ADHD. Likewise, we were unable to assess risk due to perinatal HIV exposure since all included children were exposed.

Several strengths of our study should also be noted and include the availability of in-depth neuropsychological test results performed by trained researchers and the use of a community-based (as opposed to clinical) sample. This study also incorporated a rigorous evaluation of the construct validity of a widely used DSM-IV-TR-based scale [ADHD-Rating Scale-IV (DuPaul *et al*. 1998) – which remains relevant for the unchanged DSM-5 ADHD symptom criteria (American Psychiatric Association, 2013)] using validated questionnaire and performance-based neuropsychological tests administered by trained researchers. We also included comprehensive evaluations of possible medical risk factors [including HIV treatment status, history of malaria, and nutritional status (BMI)] and family level risk factors – including the validated and widely used HOME instrument (Caldwell & Bradley, 1984).

In conclusion, our study addresses the need for valid measurement tools for global child mental health; the ADHD-RS-IV is a useful and practical tool for assessing ADHD in Sub-Saharan African settings and could be used in future epidemiologic and treatment studies. Future research efforts are needed to clarify the normative equivalence of ADHD assessment tools in SSA settings to enhance their clinical utility in settings where psychiatrists are not available. Questions also remain about the contribution of endemic risk factors for ADHD in regions like Uganda where there are high rates of malnutrition, infectious disease, and extreme poverty as well as their impact on clinical presentations of ADHD.

Our study also demonstrates higher levels of executive function deficits in Ugandan HIV-affected children who met symptom criteria for ADHD compared with those who did not meet symptom criteria for ADHD. While we were unable to assess ADHD prevalence, our finding of increased executive function problems provides supportive evidence that ADHD may be an important and impairing neurocognitive disorder among children in other similar populations in SSA. While evidence-based pharmacologic and psychosocial treatments exist for ADHD, few studies have yet targeted ADHD in low-income countries or evaluated cognitive outcomes of interventions in HIV-infected or HIV-exposed children. A recent systematic review of mental health treatments for children and adolescents in low-income countries identified only two trials targeting ADHD (both of which evaluated the efficacy of psychotropic medications) (Klasen & Crombag, 2013). Studies specifically targeting HIV treatment factors (e.g. through earlier initiation of antiretroviral therapy) have failed to improve child neurodevelopmental outcomes (Puthanakit *et al*. 2013). In contrast, an ongoing caregiver intervention trial in SSA improved overall cognitive development of preschool-age children with HIV infection though, ADHD and executive function were not specifically assessed (Boivin *et al*. 2013). Thus, there remains an urgent need to further define the burden of ADHD and to evaluate the potential impact of treatments for ADHD and related cognitive disorders among children living in SSA.
